# Assessment of Brain Natriuretic Peptide Levels in Patients Presenting to the Emergency Department With High Blood Pressure

**DOI:** 10.7759/cureus.107222

**Published:** 2026-04-17

**Authors:** Cihangir Doruk

**Affiliations:** 1 Emergency Medicine, Esenyurt Necmi Kadıoğlu Devlet Hastanesi, İstanbul, TUR

**Keywords:** antihypertensive treatment, blood pressure, brain natriuretic peptide, emergency department, hypertension

## Abstract

Objective: Hypertension is one of the leading causes of cardiovascular morbidity and mortality worldwide. Although patients presenting to emergency departments (EDs) with high blood pressure (BP) are frequently seen, it is difficult to distinguish patients with true hemodynamic or cardiac stress. Brain natriuretic peptide (BNP), secreted in response to ventricular wall strain, may serve as a useful biomarker. This study was conducted to evaluate BNP levels in patients presenting to the ED with high BP and to examine their relationship with systolic and diastolic pressures according to the antihypertensive treatment status.

Methods: This single-center, prospective study included 204 adults who presented with systolic BP ≥140 mmHg and/or diastolic BP ≥90 mmHg between July 2012 and December 2012. Patients with heart or kidney failure, acute coronary syndrome, or pregnancy were excluded from the study. Plasma BNP was measured using a chemiluminescent immunoassay. Participants were grouped according to antihypertensive medication use, and BNP levels were compared with BP values.

Results: The median BNP level was 79.4 pg/mL (range: 9.1-3500). BNP showed a moderate correlation with diastolic blood pressure (DBP) (r = 0.523; p < 0.001) but did not correlate with systolic blood pressure (SBP) or pulse rate (p > 0.05). BNP levels were higher in untreated patients than in treated patients (median: 259.5 and 67.7 pg/mL, p < 0.001). No significant difference was observed between drug classes. DBP was the only independent predictor of BNP.

Conclusion: BNP levels were significantly higher in patients not receiving regular antihypertensive treatment, particularly in those with higher DBP. BNP measurement in selected ED cases may provide additional information regarding hemodynamic stress; however, larger multicenter studies are needed to clarify its clinical utility and determine whether practical thresholds can be defined.

## Introduction

Hypertension is one of the leading causes of cardiovascular morbidity and mortality worldwide and is defined as a chronic public health problem affecting more than one-third of the adult population [1]. Characterized by persistently high blood pressure (BP) levels above normal limits, this condition is often asymptomatic for long periods and is therefore detected late in most patients. Consequently, the likelihood of developing clinical conditions leading to target organ damage, such as stroke, ischemic heart disease, congestive heart failure, peripheral vascular disease, and chronic kidney disease, increases [1]. According to the World Health Organization data, hypertension is associated with approximately 9-10 million deaths each year, a significant proportion of which are preventable, with control rates being particularly low in low- and middle-income countries [1,2].

Although the number of patients diagnosed with hypertension in population-based studies has increased, it has been reported that the rate of achieving target BP values has not yet reached the desired level. Epidemiological studies conducted in Turkey, such as the Turkish Adult Risk Factor (TEKHARF series), have shown that approximately one-third of adults are hypertensive, but BP is controlled in only one-third of those receiving treatment [3]. These findings demonstrate that hypertension is not only an individual health problem but also places a significant burden on the healthcare system and leads to economic, clinical, and workforce-related consequences.

Emergency departments (EDs) are settings where elevated BP is often first detected or worsens, and where distinguishing patients with possible hypertensive crises from those with transient or uncontrolled hypertension is frequently necessary. While some patients presenting to the ED with high BP represent true hypertensive emergencies with target organ damage, a larger proportion present with transient elevations due to pain, anxiety, medication noncompliance, or discontinuation of antihypertensive medications. Within this clinical heterogeneity, it is not always possible to determine the degree of cardiac strain based solely on BP values. Therefore, evaluating biochemical markers reflecting the heart's response to pressure and volume overload may provide additional clinical information [4].

Brain natriuretic peptide (BNP) is a peptide synthesized by ventricular cardiomyocytes and released into the circulation in response to wall tension, intraventricular pressure, or volume overload [5]. It contributes to cardiovascular homeostasis through its natriuretic, diuretic, and vasodilator effects. In clinical practice, BNP is most commonly used in the diagnosis of heart failure, assessment of its severity, and monitoring of response to treatment. However, BNP elevation may also reflect underlying structural or functional cardiac abnormalities. In acute hypertensive conditions, studies have not consistently demonstrated whether the increase in BNP is due solely to concomitant heart disease or to hemodynamic stress. Although it has long been reported that uncontrolled hypertension causes left ventricular hypertrophy and consequently BNP elevation, some series have noted that BNP levels remain within normal limits in patients with essential hypertension without structural heart disease. This inconsistency necessitates a more detailed definition of the role of BNP in hypertensive cases [5,6].

Measuring BNP levels in patients presenting to the ED with high BP may reveal the biochemical reflection of the pressure load at the time of presentation and may also show potential differences between patients who have previously been treated with antihypertensive agents and those who have not. This study was conducted to evaluate BNP levels in patients presenting to the ED with high BP, primarily to investigate the relationship between BNP levels and systolic/diastolic blood pressure (SBP/DBP) and secondarily to compare these findings according to their antihypertensive treatment history. With this approach, the study sought to explore whether there were biochemically distinct subgroups among patients presenting with similar clinical complaints.

## Materials and methods

This study was derived from a specialist thesis titled “Evaluation of Brain Natriuretic Peptide Levels in Patients Presenting to the Emergency Department with High Blood Pressure,” completed in 2012 at the emergency medicine clinic of a tertiary education and research hospital in Turkey. Data were obtained from a prospective observational clinical study conducted at the same center between July 2012 and December 2012. This was a single-center study, and consecutive eligible patients were evaluated. All patients were enrolled at the time of emergency department presentation. Written informed consent was obtained from all participants, and all data were analyzed in a blinded manner to ensure confidentiality. This study was approved by the local institutional ethics committee (approval no: 2012/14) and was conducted in accordance with the principles of the Declaration of Helsinki.

Patients aged 18 years or older with a SBP of 140 mmHg or higher and/or a DBP of 90 mmHg or higher who provided informed consent were included in the study. Patients were included based on elevated BP at presentation, regardless of whether the elevation represented transient, uncontrolled, or more severe hypertensive states. Patients with known or documented congestive heart failure, acute or chronic renal failure, acute coronary syndrome, significant infection, or systemic inflammatory disease, as well as pregnant women, were excluded from the study. These exclusion criteria were established to minimize the effect of major clinical conditions known to secondarily elevate BNP levels. As a result, a total of 204 patients were included in the study.

Demographic characteristics (age, gender), smoking status, presence of diabetes mellitus, and regular antihypertensive medication use were recorded during patient admission. BP measurements were taken using a mercury sphygmomanometer based on Korotkoff phases I and V after patients rested supine for at least 10 minutes. Before the measurement, it was ensured that there had been no heavy physical exertion, caffeine intake, or smoking in the previous 30 minutes. In patients with high BP detected in the first measurement, a second measurement was taken 10 minutes later, and the average of both readings was used for analysis.

For laboratory analysis, venous blood samples for BNP measurement were collected into EDTA-containing tubes from antecubital veins during the initial evaluation in the emergency department. The samples were immediately centrifuged at 3000 x g for 15 minutes at 4°C, and plasma aliquots were separated and stored at -70°C until the day of analysis. BNP levels were measured using a two-part “sandwich” chemiluminescent immunoassay method with the Siemens Healthcare Diagnostics ADVIA Centaur analyzer. The analytical measurement range was 2-1400 ng/L, and the total coefficient of variation was below 5%.

Patients were divided into two main groups according to antihypertensive treatment history: those not receiving regular antihypertensive treatment and those receiving regular antihypertensive treatment. Regular treatment status was determined from the medication history obtained at admission. Patients receiving treatment were further divided into six subgroups based on the type of medication: beta blockers, calcium channel blockers, angiotensin-converting enzyme inhibitors (ACEI), angiotensin receptor blockers (ARB), alpha blockers, and combination therapies containing diuretics. BNP levels, SBP and DBP, and pulse rates were compared between these groups.

Statistical analysis was performed using SPSS version 15.0 (Released 2006; SPSS Inc., Chicago, IL, USA). Numerical variables showing a normal distribution were expressed as mean ± standard deviation (SD), while variables not showing a normal distribution were expressed as median (minimum-maximum). Categorical variables were presented as percentages. The Pearson chi-square test was used to compare categorical variables; depending on the distribution, the Student t-test or Mann-Whitney U test was applied for comparisons between two groups; one-way analysis of variance (ANOVA) or the Kruskal-Wallis test was used for comparisons between multiple groups. Correlations between BNP and BP parameters were analyzed using the Spearman rho correlation coefficient. Multivariable linear regression analysis was performed, including DBP, age, gender, and diabetes mellitus. A p-value of <0.05 was considered statistically significant.

## Results

A total of 204 patients presenting to the emergency department with high BP were included in the study. Of these patients, 154 were females (74%), and 50 were males (26%), with a mean age of 58.4 ± 13.1 years. Fifty-five patients (27%) were smokers, and 50 patients (25%) had a history of diabetes mellitus. At presentation, the mean SBP was 181 ± 16 mmHg, the mean DBP was 98 ± 13 mmHg, and the mean pulse rate was 96 ± 15 beats per minute (Table 1).

**Table 1:** Demographic characteristics of patients

Variable	All patients (n = 204)
Age (years)	58.4 ± 13.1
Gender (M/F)	50/154 (26%/74%)
Diabetes mellitus, n (%)	50 (25%)
Smoking, n (%)	55 (27%)
Systolic blood pressure (mmHg)	181 ± 16
Diastolic blood pressure (mmHg)	98 ± 13
Pulse rate (beats/min)	96 ± 15

Twenty patients (10%) reported not using regular antihypertensive medication at the time of presentation. Of the remaining patients, 33 (16%) were using beta-blockers, nine (4%) were using calcium channel blockers, 41 (20%) were using ACEIs, 45 (22%) were using ARBs, 12 (6%) were taking alpha blockers, and 44 (22%) were taking combination therapies containing diuretics.

The median plasma BNP level in the overall study population was 79.4 pg/mL, ranging from 9.1 to 3500 pg/mL. This wide range suggests considerable heterogeneity in BNP values across the study population. Correlation analysis revealed a moderately positive and statistically significant relationship between BNP levels and DBP (r = 0.523; p < 0.001). No statistically significant correlation was found between BNP and SBP or between BNP and heart rate (both p > 0.05).

The median BNP level in patients not receiving regular antihypertensive treatment was 259.5 pg/mL (range: 9.1-3500), while the median BNP level in patients using antihypertensive drugs was 67.7 pg/mL (range: 9.2-2636). The difference between the two groups was statistically significant (p < 0.001). In the group not receiving regular antihypertensive treatment, the mean SBP was 175 ± 16 mmHg, the mean DBP was 98 ± 15 mmHg, and the mean pulse rate was 98 ± 15 beats per minute.

When analyzed by antihypertensive drug class, median BNP levels were as follows: 124 pg/mL (range: 9.5-1884) in patients using beta-blockers, 18 pg/mL (range: 9.2-1209) in those using calcium channel blockers, 36.2 pg/mL (range: 9.3-2472) in those using ACEIs, 50 pg/mL (range: 11.2-970) in ARB users, 48.2 pg/mL (range: 15.9-2636) in alpha blocker users, and 79.4 pg/mL (range: 10.3-329) in those receiving combination therapy including diuretics. BNP levels in all these subgroups were below the median level observed in patients not receiving regular antihypertensive treatment (Table 2).

**Table 2:** Relationship of antihypertensive therapy with BNP, SBP, DBP, and pulse rate BNP: brain natriuretic peptide; SBP: systolic blood pressure; DBP: diastolic blood pressure; ACE: angiotensin-converting enzyme; ARB: angiotensin receptor blocker

Antihypertensive treatment	BNP (pg/mL)	SBP (mmHg)	DBP (mmHg)	Pulse (beats/min)
No treatment	259.5 (9.1-3500)	175 ± 16	98 ± 15	98 ± 15
Beta-blocker	124 (9.5-1884)	180 ± 17	96 ± 16	88 ± 12
Calcium channel blocker	18 (9.2-1209)	174 ± 16	103 ± 12	91 ± 15
ACE inhibitor	36.2 (9.3-2472)	185 ± 17	109 ± 11	101 ± 15
ARB	50 (11.2-970)	181 ± 16	92 ± 14	100 ± 15
Alpha-blocker	48.2 (15.9-2636)	187 ± 18	97 ± 12	100 ± 17
Combination (including diuretics)	79.4 (10.3-329)	183 ± 15	96 ± 11	97 ± 15

BNP levels increased as DBP increased; however, no similar relationship was observed between BNP and SBP. In further analysis, DBP remained significantly associated with BNP levels, while no significant association was found with age, gender, or the presence of diabetes mellitus.

## Discussion

The data obtained in this study show that BNP levels are measurable and associated with DBP in patients presenting to the ED with high BP. The moderate positive correlation observed between BNP and DBP supports previous studies reporting that BNP increases in parallel with elevated ventricular filling pressure [7]. Previous research has shown that BNP levels are elevated in hypertensive patients with left ventricular hypertrophy or diastolic dysfunction; it is noteworthy that such a relationship was detected even after excluding patients with known heart failure [7,8].

The significantly higher median BNP in the group not receiving regular antihypertensive treatment indicates a biochemical difference between patients with and without regular BP control. The finding that this difference is more related to the presence of treatment than to a specific drug class suggests that various pharmacological groups may have similar effects on BNP levels. However, as this study is a single-center study without echocardiographic data, it is not possible to establish a definitive link between elevated BNP and structural heart changes. In addition, potentially important confounding factors such as renal function, body mass index, and duration of hypertension were not available for analysis. Similarly, as samples were taken at different times of the day, circadian variability cannot be completely ruled out; however, previous studies have reported that BNP does not show significant daily fluctuations [5,8,9].

When evaluating BNP behavior in this patient population, it is also meaningful to compare it with other cardiac biomarkers frequently used in clinical practice. NT-proBNP may better reflect chronic or subclinical cardiac load due to its longer half-life, while BNP is more sensitive to acute increases in pressure and volume [10-13]. In contrast, troponins are markers of myocardial necrosis and only rise in the presence of ischemia or myocardial injury; therefore, they do not directly indicate hemodynamic overload [14]. Echocardiography provides more detailed information through measurements such as left ventricular wall thickness, E/E′ ratio, and diastolic filling pressures; however, performing echocardiography on every patient presenting to the ED is impractical. In this context, as demonstrated in the present study, BNP measurement, which can be performed rapidly with a single blood sample, may provide additional information in distinguishing patients with different hypertensive presentations [11,12,15].

From a practical perspective in the ED, requesting a BNP test for every case of high BP is not cost-effective [11,15]. However, the findings of this study suggest that BNP measurement may be of interest in patients with markedly elevated DBP who have not previously received regular antihypertensive treatment. In such patients, BNP values exceeding 200 pg/mL were observed more frequently than in treated patients, although the clinical significance of this observation remains uncertain. While our study does not propose a definitive cutoff value, the median BNP level of 259.5 pg/mL observed in the untreated group may warrant further investigation in similar populations. Multicenter studies with larger sample sizes may help establish a practical BNP threshold for use in emergency settings.

The strengths of this study include the representation of a real patient population presenting consecutively to the ED. The relatively small number of patients not receiving regular antihypertensive treatment should be considered when interpreting group comparisons. Some limitations should also be acknowledged, including the single-center design, lack of echocardiographic data, absence of detailed information regarding the duration of hypertension, and unavailability of renal function and body mass index data. In addition, the data were collected in 2012, and this should be considered when interpreting the contemporary applicability of the findings. The study population was limited to patients presenting to the ED and may not fully represent the broader hypertensive population seen in other clinical settings. In particular, including target organ damage parameters (e.g., retinopathy, microalbuminuria, and electrocardiographic left ventricular hypertrophy) in the same model could provide a clearer understanding of the point at which BNP begins to rise. In addition, clinical outcomes such as target organ damage, hospitalization, and cardiovascular events were not evaluated in this study. Likewise, patients were not stratified according to clinical categories such as hypertensive urgency, hypertensive emergency, or incidental BP elevation, which limits clinical interpretability.

In conclusion, the data presented here suggest that BNP may reflect differences among patients presenting to the ED with high BP. However, the clinical decision-making process should not rely on a single biomarker; BP levels, treatment history, symptoms, and comorbid conditions should be evaluated together.

## Conclusions

In this study involving 204 patients who presented to the ED with high BP, plasma BNP levels were found to show a statistically significant positive correlation with DBP (r = 0.523; p < 0.001), but no significant relationship was observed with SBP or pulse rate. BNP levels in patients not receiving regular antihypertensive treatment (median: 259.5 pg/mL) were significantly higher than in treated patients (median: 67.7 pg/mL; p < 0.001), regardless of the class of medication used. These findings indicate that patients presenting with high BP may constitute a biochemically heterogeneous group and that prior treatment status is associated with BNP levels. This suggests that BNP measurement may provide additional information regarding hemodynamic stress in emergency presentations, although its clinical utility requires further study.
